# Conjugation of Gold Nanoparticles to the Anti‐IL17A Aptamer Improves Anti‐Inflammatory Effects of the Aptamer in the Experimental Imiquimod‐Induced Psoriasis

**DOI:** 10.1155/ijin/9916368

**Published:** 2025-12-09

**Authors:** Razia Khorrami, Saeideh Sadat Shobeiri, Zahra Emami, Navideh Haghnavaz, Mohammad Ali Rezaee, Safoora Pordel, Malihe Moghadam, Mojtaba Sankian

**Affiliations:** ^1^ Immunology Research Center, Faculty of Medicine, Mashhad University of Medical Sciences, Mashhad, Iran, mums.ac.ir; ^2^ Cellular and Molecular Research Center, Sabzevar University of Medical Sciences, Sabzevar, Iran, medsab.ac.ir; ^3^ Department of Medical Laboratory Sciences, Faculty of Paramedical, Kurdistan University of Medical Sciences, Sanandaj, Iran, muk.ac.ir; ^4^ Clinical Biochemistry Research Center, Basic Health Sciences Institute, Shahrekord University of Medical Sciences, Shahrekord, Iran, skums.ac.ir

**Keywords:** aptamer, gold nanoparticles, imiquimod, interleukin-17, psoriasis

## Abstract

**Introduction:**

Interleukin‐17 (IL17) plays a crucial role in the development of psoriatic plaques, making it a valuable therapeutic target. Recently, aptamers have been identified as promising candidates for inhibiting the biological activity of biomolecules. Despite these advantages, their applications are limited due to negative charge, small size, and lower affinity compared to monoclonal antibodies. To overcome these limitations, in this study, we assessed the therapeutic effects of the gold nanoparticle (AuNPs)‐conjugated anti‐IL17 single‐stranded DNA aptamer in the imiquimod‐induced C57BL/6 psoriasis animal model.

**Methods:**

Hydrogel‐containing anti‐IL17A aptamer (M2) or aptamer‐conjugated AuNPs were applied topically to the dorsal skin of the C57BL/6 mice 10 min before imiquimod treatment for 5 consecutive days. Psoriasis lesions and skin tissue sections were evaluated using the modified psoriasis area severity index (PASI) score and histology. The mRNA expression levels of inflammatory factors, including *IL17A, Interleukin1β (IL1β)*, and S100 calcium‐binding protein A9 (*S100A9*), were assessed using reverse transcription‐quantitative polymerase chain reaction.

**Results:**

Cumulative modified PASI score, as well as *IL17A, IL1β,* and *S100A9* mRNA expression levels, showed a significant decrease in the mice treated with anti‐IL17A aptamer and anti‐IL17A aptamer‐conjugated AuNPs in comparison to the imiquimod group (*p* < 0.05). The combination of anti‐IL17A aptamer with AuNPs in low concentrations (44 pmol) significantly reduced the thickness of the keratinocyte layer (*p* < 0.05). In accordance with these results, treatment with anti‐IL17A aptamer with AuNPs improved the modified PASI score in the mice skin.

**Conclusion:**

The findings of our study suggest that the anti‐inflammatory properties of anti‐IL17A (M2) aptamer are amplified when conjugated with AuNPs in the psoriasis‐like model. It seems to be a promising alternative for inhibiting the antibodies of IL17.

## 1. Introduction

Interleukin‐17 (IL17) is a crucial cytokine in the pathogenesis of psoriasis, a chronic, noninfectious inflammatory skin disease characterized by immune dysregulation [1]. The development and persistence of psoriatic lesions are primarily driven by the IL23/IL17 axis. IL23 is produced by dendritic cells, promoting differentiation and maintenance of Th17 cells, which subsequently produce IL17A, IL17F, and IL22. IL17, in turn, induces keratinocyte hyperproliferation, disrupts epidermal differentiation, and stimulates the production of antimicrobial peptides and proinflammatory cytokines (e.g., IL1β, IL6, and TNF‐α) and chemokines that recruit neutrophils and other cells, thereby creating and sustaining the chronic inflammatory loop characteristic of psoriatic plaques [2–6]. Alongside the IL23/IL17 axis, other proinflammatory cytokines, such as TNF‐α and IL22, further exacerbate psoriasis by synergistically activating keratinocytes and recruiting additional immune cells to lesion sites [7, 8].

Several biologic therapies targeting IL17 and/or its receptor have been approved for psoriasis treatment, including monoclonal antibodies such as secukinumab and ixekizumab targeting IL17A, brodalumab targeting the IL17 receptor, bimekizumab which inhibits both IL17A and IL17F, and finally the nanobody sonelokimab targeting IL17 isoforms, demonstrating significant clinical efficacy [9–14]. Despite these advances, limitations such as high production cost, potential for systemic adverse effects, and suboptimal tissue penetration highlight the need for alternative therapeutic strategies. To address this, in our previous studies, we identified anti‐IL17 aptamers with good efficacy in a psoriasis mouse model. Aptamers, single‐stranded DNA or RNA molecules, have emerged as promising candidates for inhibiting specific biomolecules due to their small size, stability, and modifiability [15, 16]. In particular, the ssDNA aptamer M2, which targets IL17A, has shown potential therapeutic effects in psoriasis models [17]. However, aptamers face challenges, including their negative charge, small size, and relatively lower binding affinity compared to monoclonal antibodies. To overcome these limitations, the conjugation of M2 ssDNA aptamer with gold nanoparticles (AuNPs) has been proposed to enhance stability, tissue penetration, and therapeutic efficacy [18].

Therefore, in this study, we aimed to evaluate the therapeutic potential of the anti‐IL17A aptamer M2 (17 nucleotides) conjugated with AuNPs in the imiquimod‐induced C57BL/6 mouse model of psoriasis. To address dose‐dependent effects, two concentrations of the aptamer, with and without AuNP conjugation, were investigated. This approach may provide a novel strategy for topical psoriasis treatment.

## 2. Materials and Methods

### 2.1. Preparation of AuNPs

#### 2.1.1. AuNP Synthesis

Spherical particles measuring 15 nm were synthesized using the Turkevich method [15]. Briefly, an aqueous solution of HAuCl4 (1 mM, 25 mL) was brought to the boil with stirring, and then trisodium citrate solution (38.8 mM, 2.5 mL) (HiMedia, India) was rapidly added to the HAuCl4. The solution was then boiled for 30 min; during this process, the colour changed from yellow to burgundy. The AuNP solution was allowed to reach room temperature before being stored at 4°C.

### 2.2. Characterizing the Physical Properties of AuNPs

The size, dispersion, zeta potential, and morphology of the synthesized AuNPs were analysed using dynamic light scattering (DLS) Zetasizer (Nano ZS, Malvern Instruments Ltd., Malvern, UK). Furthermore, scanning electron microscopy (SEM) with the MIRA3 TESCAN (Czech Republic) was employed to illustrate the morphology of the AuNPs.

### 2.3. Saturation State of Aptamer‐Conjugated AuNPs

To determine the minimum and maximum concentration range of Apt that can bind to each AuNP, we used agarose gel electrophoresis. This is a crucial factor that impacts the stability and functionality of the aptamer‐AuNPs [19].

AuNPs (2.13 pmol, 630 μL) were conjugated with various concentrations of activated M2 Apt, including 350, 175, 87.5, 44l, 22, 11, and 5.5 pmol. To perform electrophoresis, these combinations were loaded onto a 2.5% agarose gel.

### 2.4. Conjugation of AuNPs to Anti‐IL17 M2 Apt

M2 Apt with the sequence 5′‐ATA​CAA​CTG​GAT​TGT​AT‐3′ [20] was utilized in nonthiolated (SinaClon, Iran) and thiolated (MEBEP, China) forms. The thiolated form was used to conjugate the Apt to AuNPs. Briefly, the thiol groups of the Apt 50 μL (10 pmol) were activated by incubation with 5 μL dithiothreitol (DTT, 20 mM) buffered in 45 μL Tris‐HCl (10 mM, pH 7.4) at room temperature (RT) for 1 h. The solution was then washed three times with ethyl acetate to remove DTT.

For the preparation of both saturated and unsaturated AuNPs, 70 μL (350 pmol) and 8.75 μL (≈44 pmol) of thiolated Apt, respectively, were mixed with 630 μL (2.13 pmol) of AuNPs. The mixture was then incubated at RT for 24 h in the dark. Subsequently, the mixture was centrifuged at 13,400 rpm for 20 min to remove unbound Apt. To investigate the binding between Apt and AuNPs, we used 2.5% agarose gel electrophoresis.

### 2.5. Mice

Thirty five C57BL/6 female mice, weighing 17–24 g and 7–8 weeks old, were provided by the Pasteur Institute of Iran. Animals were maintained under standard conditions at 25°C and a 12‐h light–dark cycle with free access to standard mouse chow and adequate water. All experiments were conducted in accordance with the approval of the ethics committee of the Mashhad University of Medical Sciences under license number IR.MUMS.MEDICAL.REC.1401.215.

### 2.6. Psoriasis Animal Model and Experimental Groups

Mice were divided into nine groups (*n* = 4). Topical administrations were applied to the dorsal skin (2 cm^2^) of mice using an applicator. IMQ cream (40 mg) was administered in all experimental groups except Vaseline group (Vas, negative control, received 40 mg of Vas). One group received 10 mg/kg dexamethasone (Dex) through intraperitoneal injection (treatment control group) [21] and another group received 630 μL of AuNPs alone.

**Figure 1:**
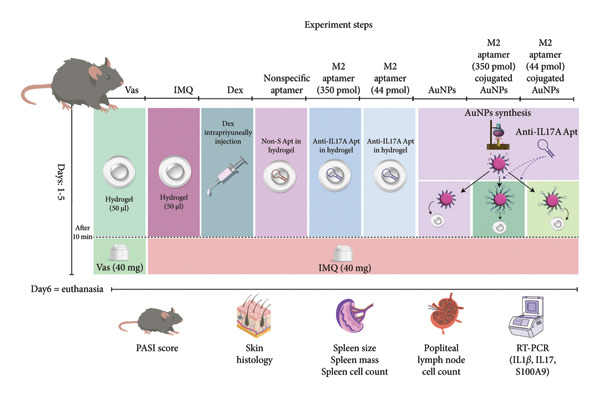
The steps of the research process. Vas: Vaseline group, IMQ: imiquimod, Dex: dexamethasone, Non‐S: nonspecific aptamer, and AuNPs: gold nanoparticles (*n* = 4, except for the Dex group, where *n* = 3).

To achieve Apt refolding, it was heated for 5 min at 95°C. It was then cooled at room temperature for 10 min [22]. The Apt was conjugated to the AuNPs before treating two groups with Apt‐conjugated AuNPs (44 and 350 pmol). In addition, two groups were treated with Apt‐unconjugated AuNPs (44 and 350 pmol). One group received 40 pmol of nonspecific (Non‐S) Apt as aptamer specificity control. All Apts were mixed in 50 μL hydrogel. The schematic picture of the method procedure is shown in Figure 1.

The mice were euthanized using carbon dioxide gas (a fill rate of 60% displacement of the chamber volume/minute) on day 6, and skin lesions were removed for histological analysis and evaluation of psoriasis‐related gene expression using reverse transcription‐quantitative polymerase chain reaction (RT‐qPCR). Furthermore, the spleen and popliteal lymph nodes of the mice were removed for analysis of total cell count, as well as weight and length of the spleen.

### 2.7. Analysis of Modified PASI Score

The mice’s weight was assessed and recorded daily during the study, and then two blinded experts scored the modified PASI score parameters—redness, scaling, and thickness—on a scale of 0–4 (0: none, 1: mild, 2: moderate, 3: severe, and 4: very severe). The cumulative modified PASI scores (0–12) were also calculated for each mouse to indicate the severity of psoriatic inflammation [23].

### 2.8. Histology

A part of the mouse back skin was collected for histological examination, fixed in 10% formaldehyde, embedded in paraffin blocks, and stained with haematoxylin–eosin (H&E). The epidermal thickness was measured for all mice by evaluating the area and length using ImageJ software version 1.44 (NIH, Bethesda, MD, USA) after observing the samples under an optical microscope (BEL Photonics, Italy). Additionally, to quantitatively evaluate histological changes, the Baker scoring system, which ranges from 0 to 10, was employed [24].

### 2.9. Total Cell Counts in the Popliteal Lymph Nodes

Popliteal lymph nodes in the legs of mice were identified by injecting 1% Evans blue dye (10 μL) into the left footpad. After 10 min, the mice were euthanized, and removal popliteal lymph nodes were dissected in 1 mL of PBS. The number of cells in the PBS (pH: 7.4) was calculated using a haemocytometer.

### 2.10. Measurement of Spleen Mass, Size, and Cell Number

The weight of each mouse’s spleen was determined in milligrams (mg) using a digital scale (A&D, Japan), and the spleen size (length) was measured in centimeters with a ruler. To collect and count the spleen cells, 1 mL of PBS was injected into one side of the spleen using a syringe. Subsequently, 1.5 mL of RBC lysis solution (NH_4_Cl, NaHCO_3_, and EDTA) was added and then centrifuged at 2000 rpm. This process was repeated, and finally, the remaining pellet was resuspended into 1 mL PBS. The number of cells was determined using a hemocytometer. The results were normalized based on the mice weight.

### 2.11. Assessment of mRNA Levels of the *S100A9*, *IL1β*, and *IL17A*

To assess gene expression, total RNA was extracted from 10 mg of the skin using a commercial kit (Pars Tous, Iran). The quality of the RNA was verified through 1% agarose gel electrophoresis. Then, reverse transcription of RNA was performed using the reverse transcriptase enzyme and oligo dT primers (Pars Tous, Iran) following the manufacturer’s instructions. The synthesized cDNA was used in a real‐time PCR to assess the expression of *IL17A, IL1β,* and *S100A9* genes.

The specific primers for *IL17A, IL1β*, and *S100A9* genes used in this study were based on our previous study [20]. Additionally, the *β2-microglubolin* gene (F primer: 5′‐GGT​CTT​TCT​GGT​GCT​TGT‐3′ and R primer: 5′‐TTC​AGT​ATG​TTC​GGC​TTC​C‐3′, product length 111 bp) was used as the housekeeping gene. PCR was conducted in duplicate/3 times using a RT‐qPCR system (Four E’s Scientific, Guangzhou, China).

### 2.12. Statistical Analysis

GraphPad Prism (version 8.4.2, California, USA) was used to analyse the data. The results were presented as mean ± standard error (SEM). Two‐way analysis of variance (ANOVA) and Mann–Whitney *U* test were used to compare the groups.

## 3. Results

### 3.1. Morphological Analysis of AuNPs

The prepared AuNP analysis indicated a relatively uniform dispersion, as evidenced by the polydispersity index (PDI) of 0.381 in the DLS results. The size and zeta potential of the prepared AuNPs were determined to be 17.6 ± 4 nm and −18 mV, respectively.

**Figure 2:**
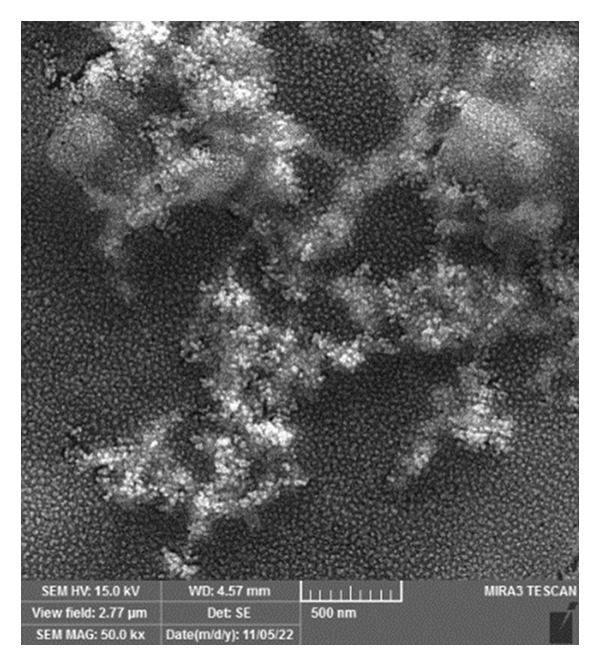
Image of AuNPs, taken with a scanning electron microscope, with a scale bar indicating a size of 500 nm.

In contrast to the unmodified AuNPs, the Apt‐conjugated AuNPs exhibited movement during electrophoresis, probably due to the introduction of negative charges by the Apt. The successful binding of the Apt to the AuNPs was further confirmed by an increase in the zeta potential to −46.6 mV. SEM results revealed that the AuNPs were uniformly dispersed and displayed a spherical shape (Figure 2).

### 3.2. Determination of the Saturation State of Aptamer‐Conjugated AuNPs

**Figure 3:**
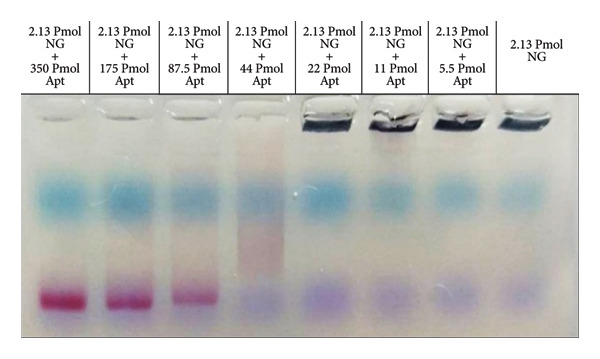
Determination of the Apt‐conjugated AuNP concentration. Electrophoresis was conducted to determine the minimum and maximum amounts of Apt required on AuNPs. Insufficient Apt leads to AuNP aggregation, while adding a sufficient amount prevents aggregation and maintains their red colour. Additionally, the negative charge of Apt causes the movement of AuNPs to be visible on the electrophoresis gel. The results indicate that the minimum and the maximum required amounts of Apt are 8.75 μL (44 pmol) and 70 μL (350 pmol), for unsaturation and saturation states, respectively.

The electrophoresis results demonstrated that the Apt‐conjugated AuNPs moved through the gel, and their red colour allowed for visual tracking with the naked eye. The minimum visible saturation and the maximum visible saturation of the Apt‐conjugated AuNPs were determined to be 8.75 μL (44 pmol) and 70 μL (350 pmol). The AuNP control demonstrated that AuNPs aggregated without Apt (Figure 3).

### 3.3. Evaluation of Modified PASI Scores

Apt‐conjugated AuNPs in two various concentrations of Apt were topically administered in the IMQ‐induced psoriasis. The IMQ group showed a significant increase in modified PASI compared to the Vas group (*p* = 0.01). Non‐S Apt group and Apt groups without AuNPs, including Apt (44 and 350 pmol), showed no significant decrease in modified PASI compared to the IMQ group. Apt‐conjugated AuNPs (44 and 350 pmol) showed a significant decrease in modified PASI compared to the IMQ group (*p* = 0.01). This enhancement in anti‐inflammatory effect was observed more significantly in the Apt‐conjugated AuNP (44 pmol) group (*p* = 0.01) (Figures 4(a), 4(b), 4(c), 4(d), 4(e)).

Figure 4The effects of Apt and Apt‐conjugated AuNPs on the skin manifestations (a). Treatment with Vaseline (Vas), IMQ cream, dexamethasone (Dex), and various doses of Apt (44 and 350 pmol) and Apt‐conjugated AuNPs (44 and 350 pmol) groups showed reduced redness (b), scaling (c), thickness (d), and modified PASI score (e) of skin lesions. The Apt‐conjugated AuNP (44 pmol) group showed a more significant improvement in clinical skin manifestations (*p* = 0.01). Because the AuNPs have red appearance, they can result in red marks on the skin, which are considered in the evaluation of redness. Non‐S: nonspecific aptamer (*n* = 4, except for the Dex group, where *n* = 3; one mouse expired due to a cause unrelated to skin issues and was thus excluded from the study). The results are presented as mean ± SEM.

**Figure:**
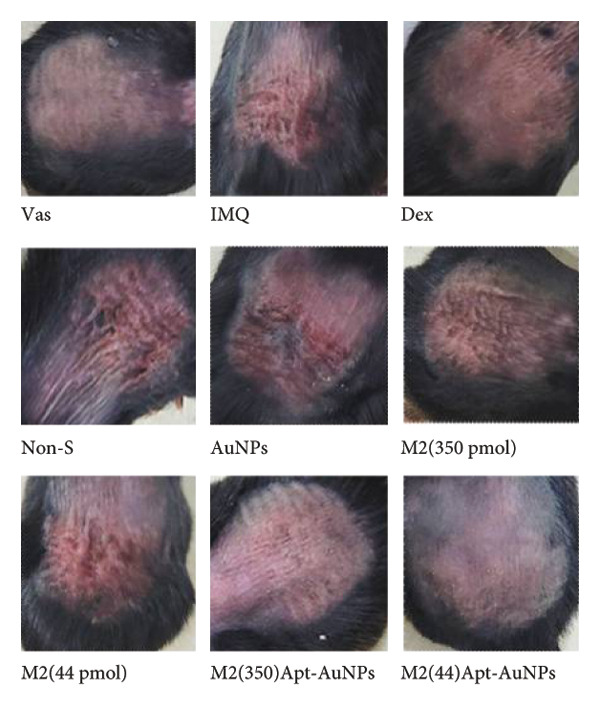
(a)

**Figure:**
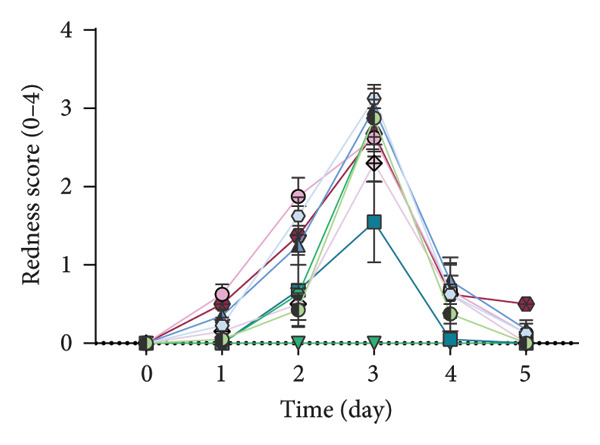
(b)

**Figure:**
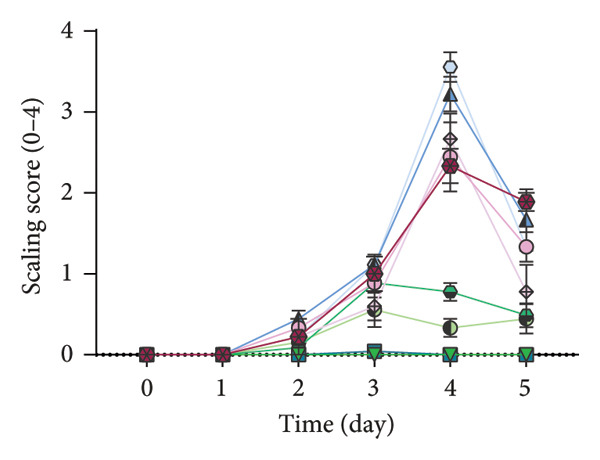
(c)

**Figure:**
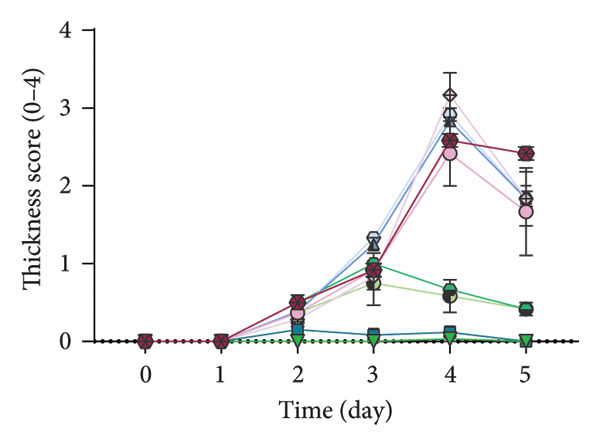
(d)

**Figure:**
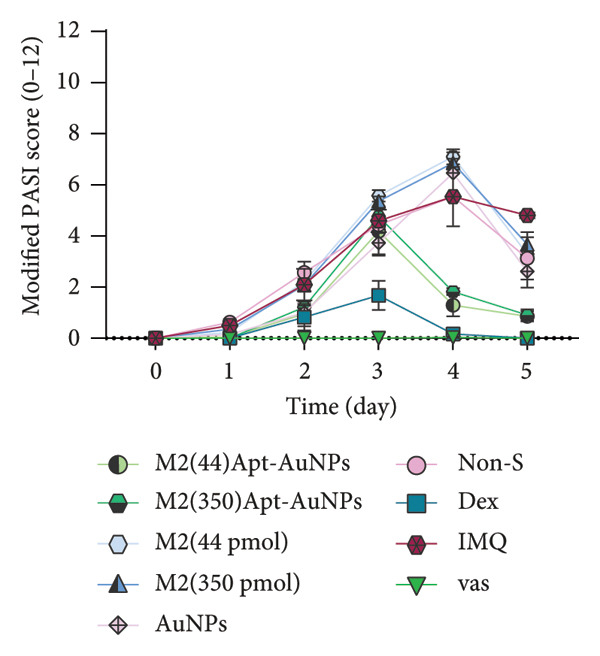
(e)

### 3.4. Systemic Effects of Aptamer and Apt‐Conjugated AuNPs

Examination of the mice weight revealed that there were no significant changes observed in any groups compared to the Vas control group (Figure 5(f)). Variations in spleen cell count, length, and mass in the IMQ group compared to the Vas group were significant only for spleen cell count (*p* = 0.02). Additionally, spleen cell counts in all groups did not exhibit a significant decrease compared to the IMQ group (Figures 5(a), 5(b), 5(c), 5(d)).

Figure 5Assessment of changes in the spleen and lymph node of C57BL/6 mice in response to administration of Apt and Apt‐conjugated AuNPs. The findings revealed an increase in the size, weight, and cell count of the spleen in the IMQ group. Parameters assessed included spleen cell count (b), spleen size (c), spleen mass (d), and LN cell count (e). Additionally, the weight of mice was assessed from 0 to 5 (f). Vas: Vaseline group, Dex: dexamethasone, Non‐S: nonspecific aptamer, and LN: lymph node (*n* = 4, except for the Dex group, where *n* = 3). All the results are presented as mean ± SEM. ^∗^ *p* < 0.05 and ^∗∗^ *p* < 0.01.

**Figure:**
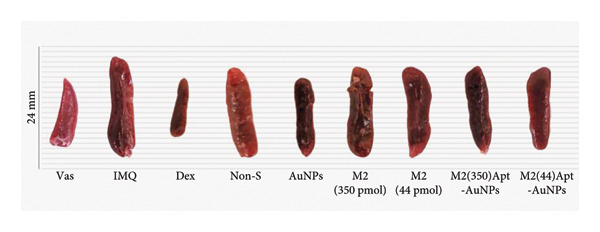
(a)

**Figure:**
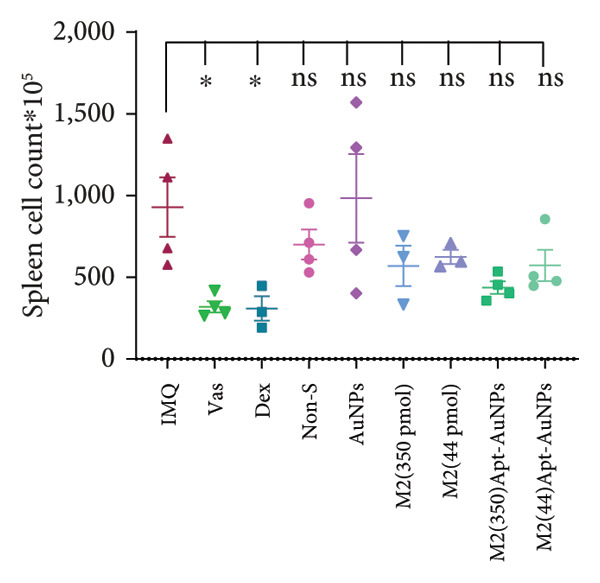
(b)

**Figure:**
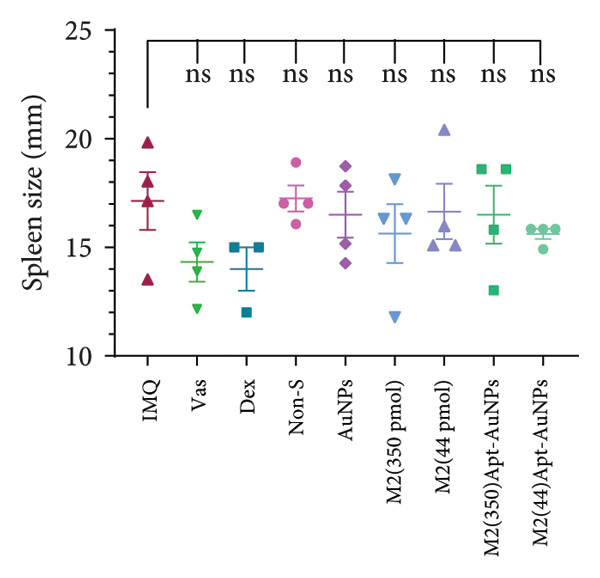
(c)

**Figure:**
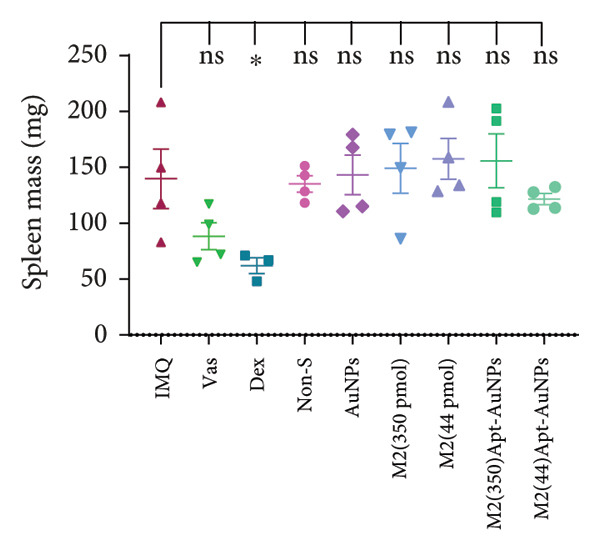
(d)

**Figure:**
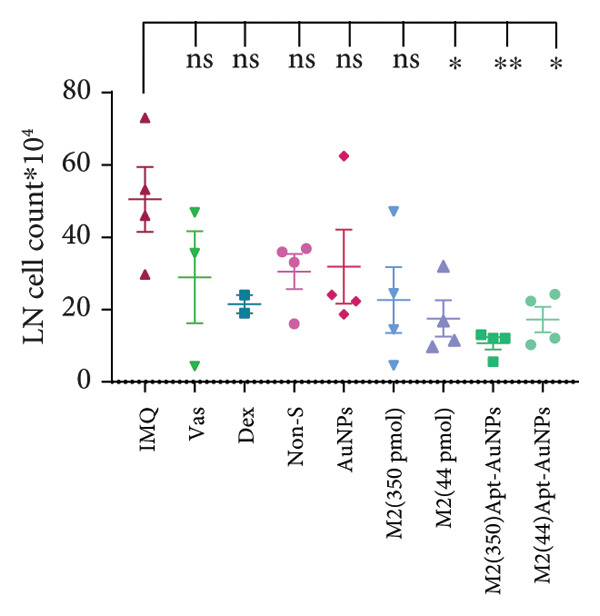
(e)

**Figure:**
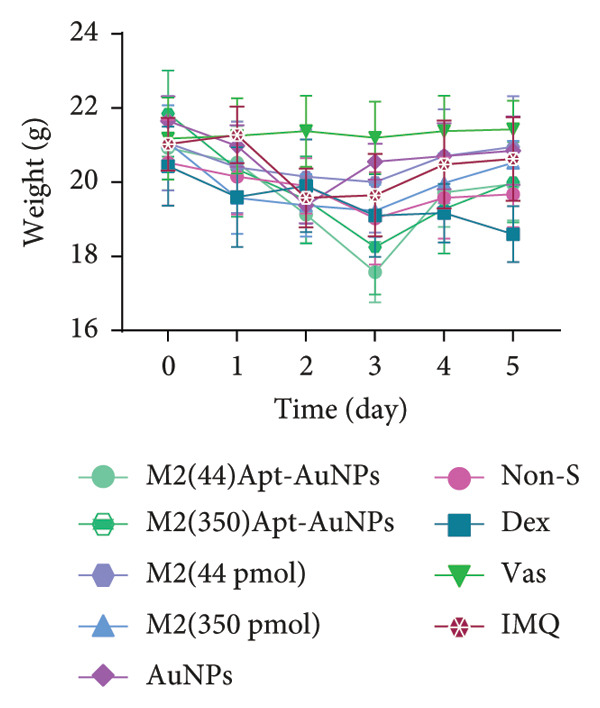
(f)

The results of counting the lymph node cells in the IMQ group showed no significant increase compared to the Vas group, while the Apt (44 pmol) (*p* = 0.04) and Apt‐conjugated AuNPs (44 and 350 pmol, in both dose *p* = 0.02) groups showed a significant decrease compared to the IMQ group. The Apt‐conjugated AuNP (350 pmol) group showed the most reduction (*p* = 0.02) in lymph node cell count (Figure 5(e)).

### 3.5. Histology

Statistical analysis of H&E‐stained tissue sections showed that the proliferation of epidermal keratinocytes in the dorsal skin of mice was significantly increased in the IMQ group compared to the Vas group (*p* < 0.0001) (Figure 6(a)). Epidermal thickness showed a significant decrease in all Apt groups including 44 (*p* = 0.002) and 350 (*p* = 0.01) pmol, Apt‐conjugated AuNPs in both doses 44 and 350 pmol (*p* < 0.0001), and AuNP alone group (*p* = 0.009), compared to the IMQ group. The reduction of epidermal thickness in the Apt‐conjugated AuNP (44 pmol) group was greater than Apt‐conjugated AuNP (350 pmol) group. Likewise, the Non‐S Apt and AuNP groups did not show a significant decrease compared to the IMQ group (Figure 6(b)).

Figure 6Histological examination of mouse skin tissue sections (H&E staining). Each part of the histological images represents a different group (a). Measurement of the epidermal thickness of the mice was conducted using ImageJ software. The score was calculated based on the width of the epidermis of each mouse (b). Quantitative assessment of histological changes using the Baker scoring system, which ranges from 0 to 10 (c). Vas: Vaseline group, IMQ group: it received the Imiquimod 5% cream, Dex: dexamethasone, Non‐S: nonspecific aptamer, and AuNPs: gold nanoparticles (*n* = 4, except for the Dex group, where *n* = 3). The results are presented as mean ± SEM. ^∗^ *p* < 0.05, ^∗∗^ *p* < 0.01, and ^∗∗∗^ *p* < 0.001.

**Figure:**
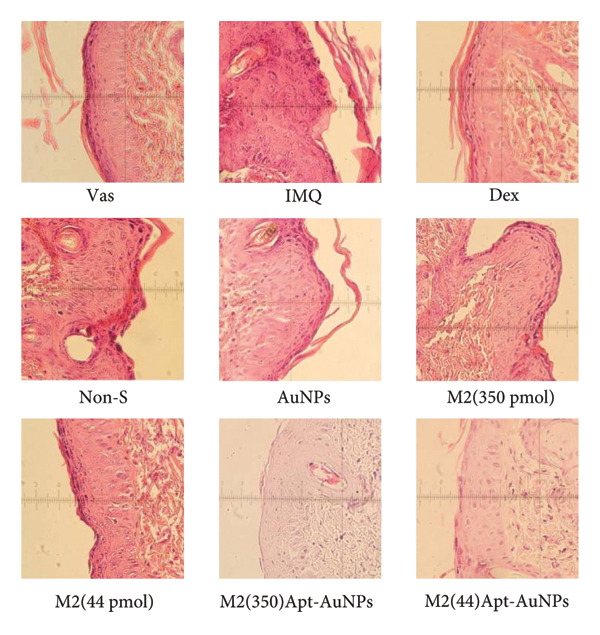
(a)

**Figure:**
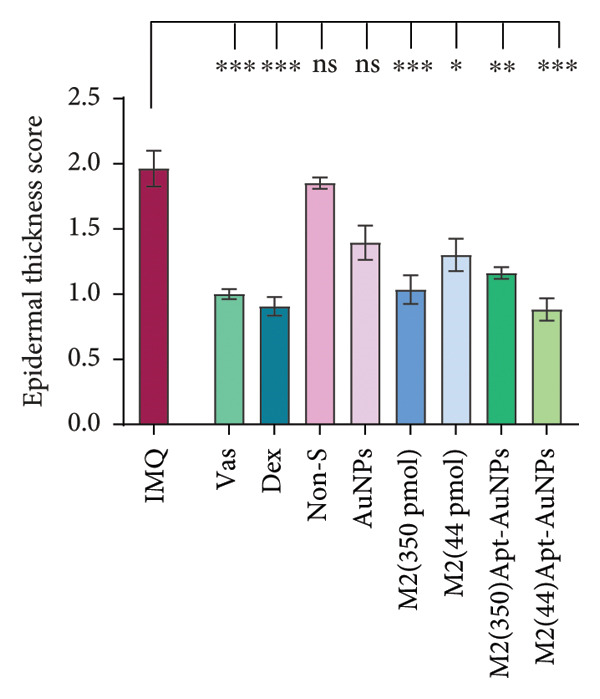
(b)

**Figure:**
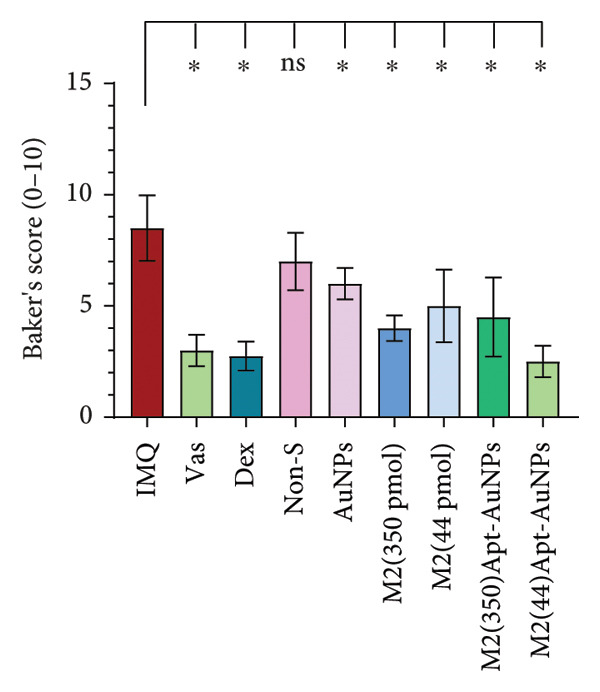
(c)

Histopathological analysis was performed using the Baker scoring method. In the Vas control group, the total Baker score was 3, while in the untreated IMQ patient control group, the Baker score was 8.5, indicating a significant increase compared to the Vas control group (*p* = 0.01). In the aptamer‐treated groups, the Baker score was significantly reduced to 5 (*p* = 0.02) in the Apt (44 pmol) group and 4 (*p* = 0.01) in the Apt (350 pmol) group compared to the IMQ control group. In the Apt‐conjugated AuNP‐treated groups, the Baker score was significantly reduced to 2.5 (*p* = 0.02) in the Apt‐conjugated AuNP (44 pmol) group and 4.5 (*p* = 0.02) in the Apt‐conjugated AuNP (350 pmol) group compared to the IMQ control group (Figure 6(c)).

### 3.6. Anti‐IL17A M2 Apt‐Conjugated AuNPs Decreased the Expression Levels of Inflammatory Cytokines

In psoriatic lesions, produced inflammatory factors are the main mediators of skin pathogenesis. *IL1β, IL17A,* and *S100A9* mRNA expression levels were measured using RT real‐time PCR. IMQ cream increased mRNA expression levels of *IL1β* (*p* = 0.05)*, IL17A* (*p* = 0.27), and *S100A9* (*p* = 0.03) in the IMQ mice skin compared to the Vas control group. The mRNA expression levels of *IL1β*, *IL17A*, and *S100A9* decreased in all treatment groups compared to the IMQ group, including the Apt (44 pmol): *IL1β* (*p* = 0.03), *IL17A* (*p* = 0.03), and *S100A9* (*p* = 0.05); the Apt (350 pmol): *IL1β* (*p* = 0.2), *IL17A* (*p* = 0.03), and *S100A9* (*p* = 0.08); Apt‐conjugated AuNPs (44 pmol): *IL1β* (*p* = 0.03), *IL17A* (*p* = 0.05), and *S100A9* (*p* = 0.02); and the Apt‐conjugated AuNPs (350 pmol): *IL1β* (*p* = 0.2), *IL17A* (*p* = 0.03), and *S100A9* (*p* = 0.05) (Figure 7).

Figure 7The administration of Apt (44 and 350 pmol) and Apt‐conjugated AuNPs (44 and 350 pmol) led to a decrease in the gene expression levels of proinflammatory cytokines IL17A, IL1β, and S100A9. The relative mRNA expression levels of *IL1β* (a), *IL17A* (b), and *S100A9* (c) were assessed using quantitative real‐time PCR. All data are reported as mean ± SEM. The experimental real‐time PCR procedures were performed twice and repeated three times. Vas: Vaseline group, IMQ: imiquimod, Dex: dexamethasone, Non‐S: nonspecific aptamer, and AuNPs: gold nanoparticles (*n* = 4, except for the Dex group, where *n* = 3). ^∗^ *p* < 0.05 and ^∗∗^ *p* < 0.01.

**Figure:**
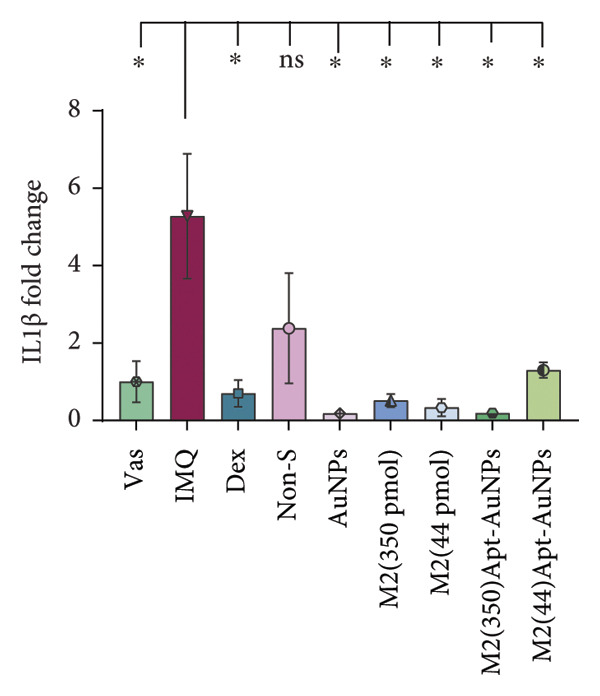
(a)

**Figure:**
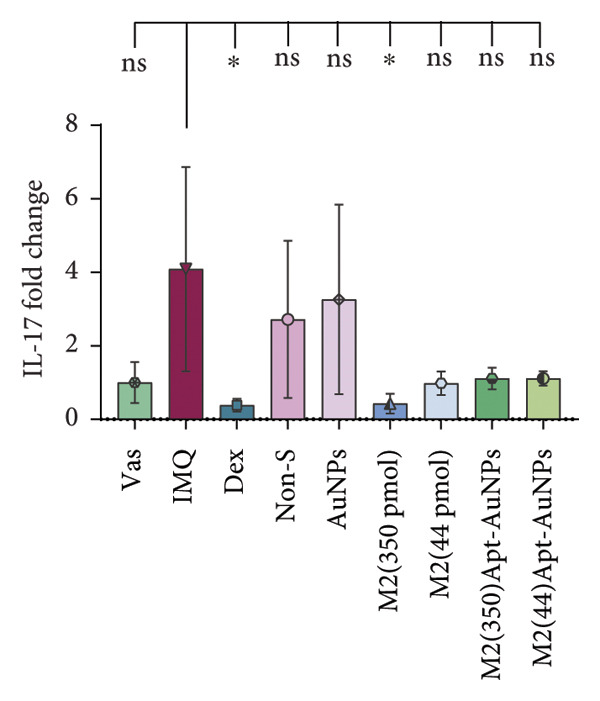
(b)

**Figure:**
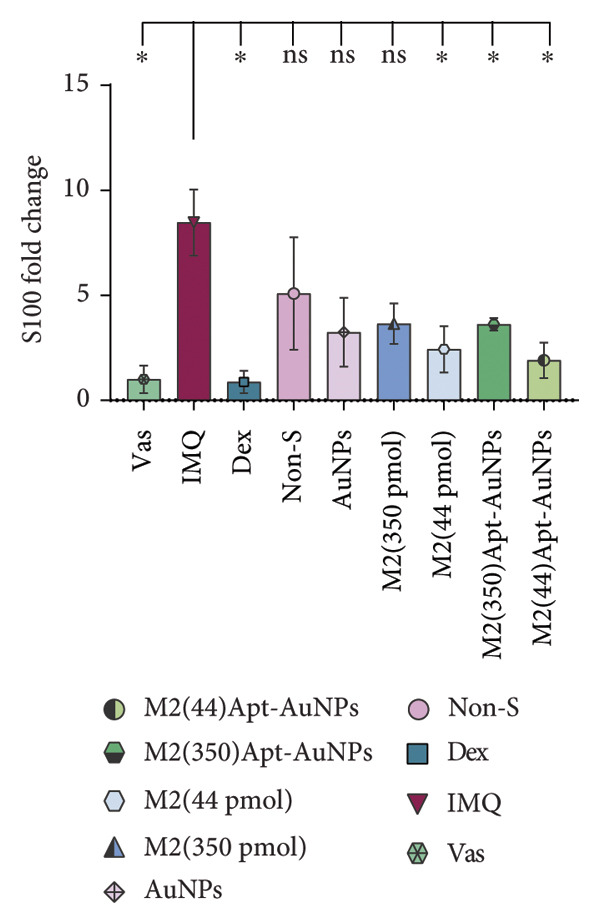
(c)

## 4. Discussion

Various strategies are used to induce psoriasis‐like symptoms and also study the development of psoriasis in mouse models, including genetic models, cytokine‐induced models, transplantation models, chemically induced models, and the commonly used IMQ‐induced model [25]. Previous studies used 62.5 mg IMQ for inducing the psoriasis mouse model [20]. In this research, 40 mg of IMQ was used in the C57BL/6 mice, successfully inducing the psoriasis mouse model, with the highest severity of psoriasis observed on the third day.

Psoriasis is an immune‐mediated condition caused by an unregulated immune response, resulting in redness, scaling, and thickening of the skin [6, 26–28]. One of the most important cells in the development of psoriasis is Th17, which produces IL17A [29] and stimulates keratinocytes, endothelial cells, and immune cells [30, 31]. Monoclonal antibodies and aptamers may be potential candidates for blocking IL17 in the psoriasis treatment [20, 32]. Aptamers as ssDNA or ssRNA structures [33] have the advantages of low immunogenicity and lower production costs compared to mABs [34]. Shobeiri et al. characterized the ssDNA Apt targeting IL17A (M7) that was effective in improving psoriasis symptoms. During an animal trial, the M2 Apt, which was smaller in size, demonstrated lower effectiveness compared to the larger M7 Apt [17, 20]. It seems that, despite the strong binding ability of M2 Apt, its smaller size may limit the ability to effectively inhibit the interaction between IL17 and IL17R. To overcome this problem, we conjugated AuNPs to M2 Apt. Nanoparticles, particularly AuNPs, are highly notable in biomedicine due to their simple synthesis and the ability to alter their physical properties like shape, size, and chemical properties as well as their combination with different materials [35]. Due to AuNP characteristics, they are employed in various applications, including drug delivery, imaging, antimicrobial and antioxidant activities, and phototherapy (PTT) [36–38]. In Joseph et al.’s study, AuNPs demonstrated immunomodulatory effects on BALB/c mice [39]. Additionally, Barreto et al.’s research indicated that the local administration of AuNPs in an atopic asthma mice model decreased Th2 proinflammatory cytokines and chemokines and prevents the primary pathological alterations [40]. Although AuNPs are generally considered biocompatible, their biological effects strongly depend on particle size, surface coating, concentration, and route of administration [41]. Several studies have demonstrated that low‐dose, surface‐functionalized AuNPs are well‐tolerated and associated with minimal systemic toxicity [42]. In contrast, repeated systemic administration or higher doses frequently result in accumulation within reticuloendothelial organs such as the liver and spleen, raising concerns about potential long‐term effects [43]. Importantly, localized or topical application substantially reduces systemic exposure and associated risks, a factor highlighted as critical in minimizing AuNP‐related toxicity [44]. This supports the rationale for their safe use in our dermatological setting.

To confirm the specificity effects of our anti‐IL17A Apt (M2), a nonspecific aptamer, which has no blocking effect on IL17A, was applied on a group of mice as Non‐S Apt control group. The results of this nonspecific aptamer‐applied group was close to the IMQ group, confirming the specificity of the anti‐IL17A Apt.

Our results showed that the experimental medication effectively alleviated psoriasis lesions, even compared with a systemic drug, dexamethasone. We used 10 mg/kg dexamethasone intraperitoneally as a positive control. Mice that received Dex had results close to the negative control group (healthy group), which showed Dex could significantly alleviate IMQ‐induced psoriatic inflammation. Salman et al. used topical 0.05% clobetasol propionate as a positive control in an imiquimod‐induced mouse model of psoriasis. Clobetasol reduced concentrations of inflammatory biomarkers, but it failed to completely improve the symptoms [45]. Li et al. found that antibodies against IL17A were less effective than dexamethasone (as positive control) in the treatment of psoriasis induced by IMQ, suggesting IL17A plays only a limited role in psoriasis pathogenesis, as dexamethasone suppressed overall inflammation [21]. Conversely, in the present study, despite the anti‐IL17 aptamer being applied topically, it demonstrated a level of improvement relatively similar to that of systemic dexamethasone treatment.

The evaluation of the spleen, popliteal lymph node, and mouse weight can be considered as factors for systemic effects of an agent. The results of our research showed an increase in the size, mass, and cell number of the spleen and popliteal lymph nodes in the IMQ group compared to the Vas group. When comparing the size, mass, and cell count between the spleen and lymph node, it was noted that the popliteal lymph node exhibits stronger reactions to the local stimulation because of its proximity to the affected area. Our results indicated no significant weight changes in any of the treatment groups, consistent with Shobeiri et al.’s findings. This indicates that neither IMQ nor Apt had a noticeable effect on the weight of the mice, and no significant systemic effect was observed [20, 46]. On the other hand, the Apt‐conjugated AuNP (low dose)‐applied mice demonstrated decrement in spleen and lymph node parameters in comparison to the Apt‐applied groups. This finding could confirm the beneficial role of AuNP conjugation for the Apt effect improving.

In the study by Peng et al., they conjugated AuNPs with the U2 DNA Apt to enhance its penetration into the brain–blood barrier (BBB) [47]. This combination successfully inhibited epidermal growth factor receptor type III and extended the lifespan of mice with glioblastoma. In consistent with this study, our results showed alleviation of psoriatic symptoms in mice which treated with Apt‐conjugated AuNPs in comparison with other groups, probably due to the enhanced penetration of Apt‐conjugated AuNPs into the skin.

In this study, we observed a decrease in the modified PASI score in all treatment groups, with a significant decrease in the Apt‐conjugated AuNP groups compared to the Apt groups. Also, we observed a decrease in the level of S100A9, IL1β, and IL17 gene expression in both specific Apt and specific Apt‐conjugated AuNPs (in two concentrations) receiving groups compared to the IMQ control group. Among them, the cases in which we conjugated AuNPs to the Apt have performed better in low concentrations (44 pmol). The high dose of Apt may be less effective, as the extra negative charge and also possible destructed DNA could lead to inflammation [48].

The assessment of IL1β expression in the group treated with AuNPs alone showed a reduction in the level of IL1β compared to other groups. In this context, Vadim et al. investigated the effect of AuNPs on inflammatory reactions induced by IL‐1β. Their results revealed that AuNPs can interact with extracellular IL‐1β, resulting in a decrease in inflammatory responses triggered by this cytokine [49]. Therefore, based on our findings, AuNPs can reduce the level of IL1β.

One of the most important factors in the evaluation of psoriasis is the examination of the epidermal thickness. In our experiment, all of the specific Apt‐receiving mice (Apt and Apt‐conjugated AuNPs in both doses) demonstrated enhancement in epidermal thickness. Among them, the cases in which we conjugated AuNPs to the Apt performed better in low concentration (44 pmol), supporting the modified PASI and gene expression results. Additionally, psoriasis symptoms in mouse skin and epidermal thickness graphs support the results. In line with our results, Fratoddi et al.’s study showed that the combination of AuNPs with methotrexate decreased the epidermal thickness in mice with IMQ‐induced psoriasis [50]. As expected, AuNPs alone and the nonspecific Apt control group did not affect clinical skin manifestations and epidermal thickness. Among the groups treated with Apt conjugated to AuNPs, the lower dose is more effective in attenuation of psoriatic inflammation. It seems this improvement could be attributed to the larger size of Apt and its enhanced steric hindrance between IL17 and IL17R. The evaluation of two different concentrations of M2 Apt conjugated to AuNPs, along with the pathological results, showed that the lower concentration of Apt‐conjugated AuNPs not only leads to more improvement but also offers more cost‐effectiveness and potentially fewer side effects.

In comparison with current therapeutic strategies targeting IL17, such as monoclonal antibodies, nanobodies, and oral inhibitors, our findings highlight several potential advantages of aptamer–AuNPs. Monoclonal antibodies, including secukinumab and ixekizumab, demonstrate strong clinical efficacy but are limited by high cost, parenteral administration, and risk of systemic immunosuppression [51]. Nanobodies, such as sonelokimab, provide improved tissue penetration and reduced immunogenicity [12, 52] but remain relatively expensive and require injection. Oral IL17 pathway inhibitors (e.g., TYK2 inhibitors such as deucravacitinib) are currently under clinical development, and while they show promising efficacy, they pose risks of gastrointestinal side effects and systemic exposure [53, 54]. In contrast, our findings suggest that aptamer–AuNPs, when applied topically, provide a localized therapeutic effect. Given their simpler synthesis and effectiveness at low doses, aptamers are expected to be more cost‐effective than monoclonal antibodies, making them a potentially safer and more accessible alternative in psoriasis management.

The imiquimod‐induced psoriasis model, while widely used and accessible for comparative studies with human psoriasis, presents several inherent limitations. These constraints may hinder the comprehensive understanding of disease mechanisms and therapeutic outcomes. Consequently, employing an IL23‐induced psoriasis model could offer significant advantages, as it directly activates the IL23/IL17 pathway, which plays a central role in psoriasis pathogenesis. Moreover, the limitations of the current study give several opportunities for future studies, particularly in the context of clinical study protocols and the rigorous analysis of key signalling pathways. Specifically, future studies must prioritize the definition of the precise roles and regulation of key signalling mechanisms like nuclear factor kappa B (NF‐κB) and signal transducer and activator of transcription 3 (STAT3) [55]. These pathways are particularly interesting since they are characterized by overexpression in psoriatic skin lesion, something which warrants an understanding and potentially targeted treatment. A greater understanding of these signal transduction processes may yield valuable data for the design of more effective treatments for psoriasis.

## 5. Conclusion

The present study showed that the combination of AuNPs with specific anti‐IL17A Apt (M2) in an IMQ‐induced psoriasis mouse model boosted the anti‐inflammatory effect of anti‐IL17A Apt, especially in low dose, and alleviated the symptoms of psoriasis in the back skin of C57BL/6 mice.

## Ethics Statement

The ethics committee of the Mashhad University of Medical Sciences approved the study protocol (ID: IR.MUMS.MEDICAL.REC.1401.215).

## Conflicts of Interest

The authors declare no conflicts of interest.

## Author Contributions

Razia Khorrami: investigation, collection and assembly of data, data analysis and interpretation, manuscript drafting, and manuscript editing. Saeideh Sadat Shobeiri: conceptualization, methodology, investigation, and manuscript editing. Zahra Emami: investigation, manuscript editing, and graphic designing. Navideh Haghnavaz, Mohammad Ali Rezaee, and Safoora Pordel: investigation and manuscript editing. Malihe Moghadam: investigation. Mojtaba Sankian: conceptualization, methodology, supervision, project administration, and manuscript editing.

## Funding

This work was supported by Mashhad University of Medical Sciences, 4001903.

## Data Availability

The data that support the findings of this study are available from the corresponding author upon reasonable request.
